# Horizontal Transfer of a Retrotransposon from the Rice Planthopper to the Genome of an Insect DNA Virus

**DOI:** 10.1128/JVI.01516-18

**Published:** 2019-03-05

**Authors:** Qiankun Yang, Yan Zhang, Ida Bagus Andika, Zhenfeng Liao, Hideki Kondo, Yanhua Lu, Ye Cheng, Linying Li, Yuqing He, Yujuan He, Yuhua Qi, Zongtao Sun, Yuanhua Wu, Fei Yan, Jianping Chen, Junmin Li

**Affiliations:** a Plant Protection College, Shenyang Agricultural University, Shenyang, China; b Institute of Plant Virology, Ningbo University, Ningbo, China; c The State Key Laboratory Breeding Base for Sustainable Control of Pest and Disease, Key Laboratory of Biotechnology in Plant Protection of MOA of China and Zhejiang Province, Institute of Virology and Biotechnology, Zhejiang Academy of Agricultural Sciences, Hangzhou, China; d Central Laboratory of Zhejiang Academy of Agricultural Sciences, Zhejiang Academy of Agricultural Sciences, Hangzhou, China; e Institute of Plant Science and Resources, Okayama University, Kurashiki, Japan; f College of Horticulture and Plant Protection, Yangzhou University, Yangzhou, China; University of Illinois at Urbana Champaign

**Keywords:** horizontal transfer, invertebrate iridescent virus 6, iridovirus, rice planthoppers, SINE, transposable element, piRNAs

## Abstract

Horizontal transfer of genetic materials between virus and host has been frequently identified. Three rice planthoppers, Laodelphax striatellus, Nilaparvata lugens, and Sogatella furcifera, are agriculturally important insects because they are destructive rice pests and also the vector of a number of phytopathogenic viruses. In this study, we discovered that a small region (∼300 nucleotides [nt]) of the genome of *invertebrate iridescent virus* 6 (IIV-6; genus *Iridovirus*, family *Iridoviridae*), a giant DNA virus that infects invertebrates but is not known to infect planthoppers, is highly homologous to the sequences present in high copy numbers in these three planthopper genomes. These sequences are related to the short interspersed nuclear elements (SINEs), a class of non-long terminal repeat (LTR) retrotransposons (retroposons), suggesting a horizontal transfer event of a transposable element from the rice planthopper genome to the IIV-6 genome. In addition, a number of planthopper transcripts mapped to these rice planthopper SINE-like sequences (RPSlSs) were identified and appear to be transcriptionally regulated along the different developmental stages of planthoppers. Small RNAs derived from these RPSlSs are predominantly 26 to 28 nt long, which is a typical characteristic of PIWI-interacting RNAs. Phylogenetic analysis suggests that IIV-6 acquires a SINE-like retrotransposon from S. furcifera after the evolutionary divergence of the three rice planthoppers. This study provides further examples of the horizontal transfer of an insect transposon to virus and suggests the association of rice planthoppers with iridoviruses in the past or present.

**IMPORTANCE** This study provides an example of the horizontal transfer event from a rice planthopper genome to an IIV-6 genome. A small region of the IIV-6 genome (∼300 nt) is highly homologous to the sequences presented in high copy numbers of three rice planthopper genomes that are related to the SINEs, a class of retroposons. The expression of these planthopper SINE-like sequences was confirmed, and corresponding Piwi-interacting RNA-like small RNAs were identified and comprehensively characterized. Phylogenetic analysis suggests that the giant invertebrate iridovirus IIV-6 obtains this SINE-related sequence from *Sogatella furcifera* through a horizontal transfer event in the past. To the best of our knowledge, this is the first report of a horizontal transfer event between a planthopper and a giant DNA virus and also is the first evidence for the eukaryotic origin of genetic material in iridoviruses.

## Abstract (precis)

This study provides an example of the horizontal transfer event from a rice planthopper genome to an IIV-6 genome. A small region of the IIV-6 genome (∼300 nt) is highly homologous to the sequences presented in high copy numbers of three rice planthopper genomes that are related to the SINEs, a class of retroposons. The expression of these planthopper SINE-like sequences was confirmed, and corresponding Piwi-interacting RNA-like small RNAs were identified and comprehensively characterized. Phylogenetic analysis suggests that the giant invertebrate iridovirus IIV-6 obtains this SINE-related sequence from *Sogatella furcifera* through a horizontal transfer event in the past. To the best of our knowledge, this is the first report of a horizontal transfer event between a planthopper and a giant DNA virus and also is the first evidence for the eukaryotic origin of genetic material in iridoviruses.

## INTRODUCTION

Horizontal transfer (HT) of genetic material has been increasingly discovered between different viruses and their eukaryotic hosts, and it shapes the evolution of the viruses and their hosts (1). During long evolution of the virus-host relationship, HT events can occur in two opposite ways: from host to virus or from virus to host. For host-to-virus HT, the viral genome can acquire various host genes, such as ubiquitin (2), chloroplast protein (3), and heat shock protein (4), during evolution. Giant viruses or nucleocytoplasmic large DNA viruses have a very large linear or circular genomic double-stranded DNA (dsDNA) molecule between 100 kb (such as some phycodnaviruses and iridoviruses) and 2.5 Mb (such as pandoraviruses). It has been reported that giant viruses contain high proportions (at least 10%) of host-derived genes, and some of these genes are key factors for viral pathogenesis (5–7). For the virus-to-host direction, viruses hijack many of the host cellular functions to facilitate their own replication, and the sequences of many viruses have occasionally been integrated into host chromosomes during these interactions, a process called endogenization (8). These integrated viral sequences, which may be whole or partial, are referred to as endogenous viral elements (EVEs) (9). With the sequencing of many eukaryotic genomes and advances in bioinformatics, many EVEs derived from retroviral or nonretroviral viruses have been discovered in a variety of eukaryotes (10). Since EVEs are integrated into the germ line and are vertically inherited in their hosts, they serve as viral imprints (fossils) and provide unprecedented opportunities to explore the evolution of viruses and their interactions with various hosts (8). Recent studies have also shown that EVEs derived from nonretroviral viruses can act as templates for the production of PIWI-interacting RNAs (piRNAs; 24 to 32 nucleotides [nt] in length), a small RNA class that was associated with Piwi-subfamily proteins, which might play essential roles in antiviral immunity of the mosquito Aedes aegypti, thereby providing a memory reservoir of past immunity events (9, 11, 12).

Besides HT events between different viruses and their eukaryotic hosts, eukaryote-to-eukaryote HT are also prevalent in nature (13). Recent studies indicated that most of the eukaryote-to-eukaryote HTs are related to transposable elements (TE), and viruses are major vectors of HT between eukaryotes (14). Piskurek et al. (15) reported that poxviruses (family *Poxviridae*) are possible vectors for HT of retroposons (a class of non-long terminal repeat [LTR] retrotransposon, subfamilies of short interspersed elements, or SINEs) from reptiles to mammals. Another example is that baculovirus (Autographa californica multiple nucleopolyhedrovirus, family *Baculoviridae*) infection facilitates HT of two transposable elements from cabbage looper (*Trichoplusia ni*) between several sympatric moth species (16). With the large amounts of new genomes and short read archives deposited in public databases, more virus-mediated eukaryote-to-eukaryote HT will no doubt be revealed and contribute to our understanding of mechanisms underlying HT between eukaryotes.

The small brown planthopper (SBPH; Laodelphax striatellus), brown planthopper (BPH; Nilaparvata lugens), and white-backed planthopper (WBPH; Sogatella furcifera), generally called rice planthoppers, belong to family *Delphacidae* (order *Hemiptera*) and are three of the most destructive insect pests of rice in tropical and temperate regions of Asia (17). In addition to direct feeding damage, they act as efficient vectors of plant viruses and phytoplasmas, including at least 18 important phytopathogenic rice viruses, some of which replicate in their vector as well as in the host plant, such as *Rice black-streaked dwarf virus* (RBSDV, a reovirus) and *Rice stripe tenuivirus* (RSV, a tenuivirus) for L. striatellus (18, 19), *Rice ragged stunt virus* (a reovirus) and *Rice grassy stunt virus* (a tenuivirus) for N. lugens (20), and *Southern rice black-streaked dwarf virus* (SRBSDV, a reovirus) for S. furcifera (21). Insect-specific viruses are also commonly reported in rice planthoppers, including *Himetobi P virus* (HiPV), a picorna-like virus that infects the three rice planthoppers asymptomatically with high frequency (22, 23). There has been little reported work on HT in rice planthoppers, except for the identification of nudivirus (family *Nudiviridae*, closely related to polydnavirus)-like sequences in the N. lugens genome. Nudivirus sequences were widely found in the scaffolds or contigs of the N. lugens genome, and these viral sequences were reported to be expressed in different tissues of the insect. However, although the rod-shaped nudivirus virions were not detected in various insect tissues by electron microscopy, the current evidence does not rule out the possibility that these integrated viral sequences are free virus in N. lugens rather than ancient viral relics (24).

Chilo iridescent virus is classified as *Invertebrate iridescent virus 6* (IIV-6), the type species of the genus *Iridovirus*, family *Iridoviridae* (25). It was originally isolated from diseased larvae of the rice stem borer (Chilo suppressalis) and has been used as the standard model for studies on invertebrate iridoviruses (26, 27). Although IIV-6 can infect more than 100 insect species belonging to at least six orders, including *Hemiptera* (leafhoppers) (27, 28), it has never been reported to infect planthoppers. Because the virus causes limited mortality to insects and has a large genome, it has received little research attention (29). Its dsDNA genome has 212,482 bp and contains 468 open reading frames (ORFs) (30, 31). Although the viruses in the family *Iridoviridae* have relatively large genome sizes, iridoviruses seem to be less prone to lateral gene exchange with their host than other giant viruses, such as poxviruses (family *Poxviridae*) and a marseillevirus (family *Marseilleviridae*) (6). In addition, eukaryotic class II DNA transposons (miniature inverted-repeat transposable elements, or MITEs) were recently identified in the genomes of iridoviruses (*Invertebrate iridescent virus 9*, IIV-9, and *Invertebrate iridescent virus 22*, IIV-22), indicating that these viruses act as vectors for HT of transposable elements between host species (32). Nevertheless, the origins of these transposons in the genome of iridoviruses are still unclear.

In this study, potential HT events of genetic material between three rice planthoppers and virus genomes were investigated. Interestingly, a small region of the IIV-6 genome (∼300 nt) is highly homologous to the sequences present in high copy numbers in rice planthopper genomes that have a sequence relatedness to SINE retroposons. Phylogenic analysis indicated that this SINE-like element is transferred from the planthopper to the IIV-6 genome in the past after the evolutionary divergence of the three rice planthoppers.

## RESULTS AND DISCUSSION

### Identification of VLSs in the genomes of three rice planthoppers.

The availability of recently published genomes of L. striatellus, N. lugens, and S. furcifera provides resources to identify virus-like sequences (VLSs) in rice planthoppers (33–35). By homology search using planthopper genomes to NCBI virus RefSeqs, 1,699, 5,422, and 4,038 VLSs were discovered in the genomes of L. striatellus, N. lugens, and S. furcifera, respectively (see File S1 in the supplemental material). Interestingly, all identified VLSs were homologous to viruses that have never been reported to infect planthoppers, and none of these viruses were from known planthopper-transmitted rice viruses (such as RSV and RBSDV) or insect-specific viruses (such as HiPV). This contrasts with recent results showing that the genome of mosquitos (major vectors of flaviviruses such as yellow fever virus and dengue virus) contains endogenous ﬂaviviral elements (36–38). Although the VLSs that we identified are similar to those of viruses that are not known to infect rice planthoppers, they might have infected planthoppers in the past and provide persistent viral fossil evidence in the host genome.

### Iridovirus-like sequence that is homologous to the sequences with high copy numbers in rice planthopper genomes.

**FIG 1:**
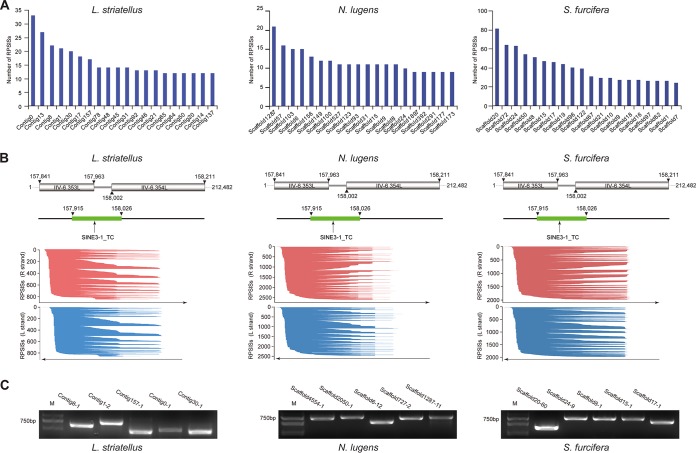
Identification of sequences homologous to IIV6_300 sequence (RPSlSs) in three planthopper genomes. (A) Bar plots showing the number of RPSlSs within contigs/scaffolds (top 20) of three planthopper genomes. (B) Coverage plots of RPSlSs mapped to the region between the ORFs 353L and 354L of the IIV-6 genome. Each line represents a single RPSlS, and its length and position denote the region of the indicated ORF to which its sequence is mapped. Red lines indicated RPSlSs mapped to the R (+) strand of IIV-6, and blue lines represents those to the L (−) strand. (C) Genomic PCR detection of five randomly selected contigs/scaffolds containing RPSlSs in three planthopper genomes.

Intriguingly, we found that the vast majority of VLSs in planthoppers were homologous to a region in the IIV-6 (an iridovirus) genome. The percentages of VLSs that are homologous to the IIV-6 sequence were 97.76%, 92.23%, and 98.41% in L. striatellus, N. lugens, and S. furcifera, respectively. The genomes of the three planthoppers next were searched against the IIV-6 genome (NC_003038.1) to confirm the presence of VLSs that are homologous to IIV-6 (File S2). Our results indicated that 1.54% of L. striatellus contigs (587/38,193), 5.34% of N. lugens scaffolds (2,485/46,559), and 4.85% of S. furcifera scaffolds (991/20,450) contain at least one sequence homologous to IIV-6 with significant matches (Table 1). The top 20 contigs/scaffolds that contain the highest numbers of homologous sequences in the three rice planthoppers are shown in Fig. 1A. IIV-6 has a large genome, and the first (so far the only) complete genome was sequenced in 2001 (30). It is 212,482 bp long and has 468 predicted ORFs (30). Surprisingly, mapping results indicated that all of the discovered homologous sequences (1,686 for L. striatellus, 5,031 for N. lugens, and 3,986 for S. furcifera), except one of N. lugens in scaffold 137, mapped to a short region (∼300 nt) from nt 157,843 to 158,142 nt of the viral genome that covered most regions of ORF 353L, the intergenic region, and parts of ORF 354L (here this region is referred to as IIV6_300) (Fig. 1B). ORFs 353L and 354L are both on the complimentary strand of the IIV-6 genome; ORF 354L encodes a protein with a predicted l-lactate dehydrogenase active site domain, while the function of 353L is currently unknown (30). The majority of the homologous sequences were only 100 to ∼200 bp long, and their integrations are almost equal in both directions (Table 1 and Fig. 1B). To experimentally validate the presence of the homologous sequences, five sequences from different contigs/scaffolds (approximately 700 bp) of each of the three planthoppers were randomly selected and amplified by PCR. Amplification products with the expected sizes were obtained from all of the selected contigs/scaffolds, and Sanger sequencing of the purified DNA products confirmed their identity (Fig. 1C). Although IIV-6 has a broad host range and can infect more than 100 insect species (27), to the best of our knowledge, this is the first report of an HT event of the genetic material between IIV-6 and a eukaryotic host.

**TABLE 1:** Summary of IIV6-LS identified in three planthopper genomes^*a*^

Species	Total no. of scaffold/contigs	No. (%) of IIV6-LS matched redundant	No. (%) of IIV6-LS matched unique	No. of matched IIV-6 genome regions	IIV-6 ORFs containing IIV6-LS mapped region	Orientation (sense/antisense)	Avg no. of matched IIV6-LS per scaffold/contig
L. striatellus	38,193	1,686 (4.41)	587 (1.54)	157,843-158,135	353L, 354L	835/851	2.872 ± 3.508
N. lugens	46,559	5,031 (10.81)	2,485 (5.34)	157,851-158,142	353L, 354L	2,579/2,452	2.024 ± 1.748
S. furcifera	20,450	3,986 (19.49)	991 (4.85)	157,844-158,137	353L, 354L	1,932/2,054	4.022 ± 6.791

a IIV6-LS, IIV6-like sequences.

### IIV6_300 sequence is a predicted transposable element of rice planthoppers.

Transposable elements are the major components of eukaryotic genomes and account for approximately 25.7%, 38.9%, and 32.6% of sequences in the genomes of L. striatellus, N. lugens, and S. furcifera, respectively (33–35). Transposable elements are pieces of DNA that are able to jump from one locus to another in the genome of their host, and the majority of HT events reported until now are the transfers of transposable elements (14). Due to the high copy numbers of the sequences that are homologous to the IIV6_300 sequence in the planthopper genomes, these sequences, including a 500-nt extension in both 5′ and 3′ termini, were analyzed for the presence of transposable element motifs using CENSOR (39). The analysis indicated that they contain the conserved SINE3-1_TC motif, which is also present in the IIV6_300 sequence (Fig. 1B). Thus, they may be short interspersed nuclear elements (SINEs), which is a class of non-LTR retrotransposon (retroposon) present in various eukaryotic genomes. Note that we did not find the rice planthopper SINE-like sequences (RPSlSs) in the genome of the rice stem borer, the known host of IIV-6. Taken together, these observations suggest that IIV-6 probably obtained a transposable element from a planthopper through an HT event. In the case of other iridoviruses, IIV-9 and IIV-22 were predicted to contain eukaryotic DNA transposon MITEs, which might result from HT (32), but the origins of the predicted eukaryotic MITEs are still unclear.

### Transcription and integration profile of RPSlSs in rice planthoppers.

**FIG 2:**
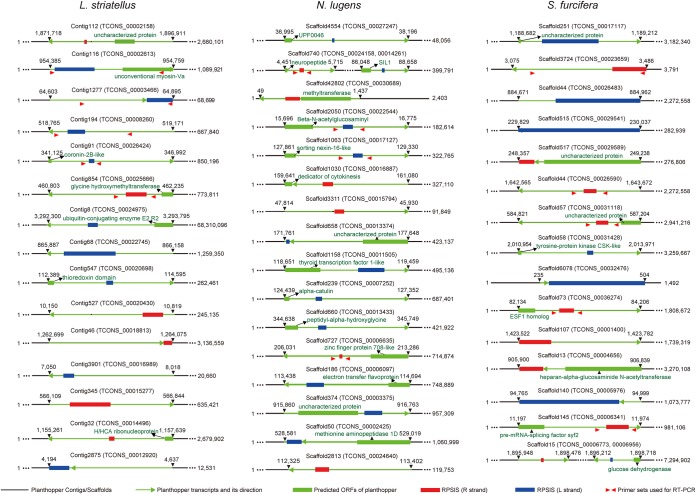
Distribution of RPSlSs within planthopper transcripts and genomes. Black lines indicate contigs/scaffolds of the planthopper. Green lines with arrows indicate transcripts of planthoppers and their direction. Green boxes represent the predicted ORF within insect transcripts. The annotation of the predicted ORF is indicated above. Red and blue boxes represent the transcripts of RPSlSs with R and L strands, respectively. Red arrow pairs indicate primer sets used for RT-PCR detection of RPSlSs.

**FIG 3:**
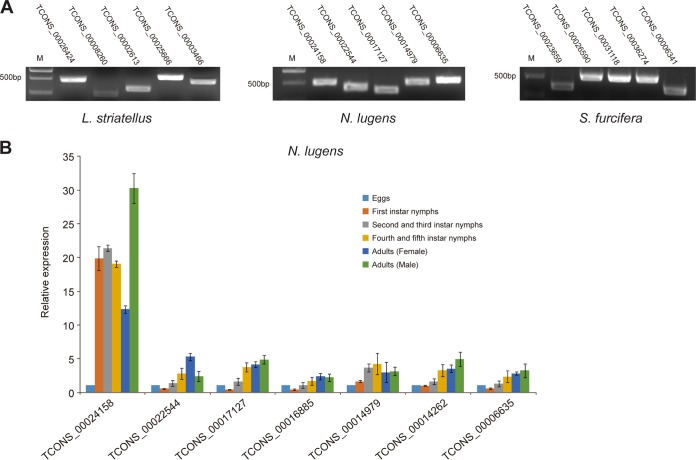
Expression of planthopper RPSlSs. (A) RT-PCR detection of planthopper transcripts containing RPSlSs. (B) Expression of RPSlSs at different developmental stages of N. lugens. The 18S gene of N. lugens was used as an internal control for normalization. Error bars represent the standard deviations using three replicates.

The complete genome of IIV-6 was used as a database and searched with the newly reassembled transcriptomes of the three planthoppers. A total of 19, 24, and 178 planthopper transcripts containing RPSlSs were found in L. striatellus, N. lugens, and S. furcifera, respectively, indicating that some of the RPSlSs are transcribed in planthoppers (Tables 2 and 3 and Table S1). As shown in Fig. 2, some RPSlSs were distributed in the transcribed regions of planthopper genes with various predicted functions, such as glycine hydroxymethyltransferase and ubiquitin-conjugating enzyme in L. striatellus, methyltransferase and electron transfer flavoprotein in N. lugens, and tyrosine-protein kinase and glucose dehydrogenase in S. furcifera. Planthopper transcripts contain RPSlSs derived from both strands (Fig. 2). In addition, five RPSlSs from each planthopper were randomly selected and analyzed by reverse transcription-PCR (RT-PCR) (Fig. 3A), followed by Sanger sequencing. The positions of the primer sets are indicated by red arrows below the transcripts (Fig. 2). The result confirmed that RPSlSs are indeed expressed in planthoppers rather than contaminant sequences from incidental exogenous sources.

**TABLE 2:** RPSlS-containing transcripts identified in assembled L. striatellus (SBPH) transcriptome

ID^*a*^	Transcriptome assembly ID^*b*^	GenBank accession no.^*c*^	Orientation	Length (nt)	E value	Match coordinate				Annotation^*d*^
						mRNA position		IIV-6 genome position		
						Start	End	Start	End	
IIV6-SBPH-1	TCONS_00002158	XP_022186857	+	254	7.00E−69	8922	9171	157870	158120	Uncharacterized protein LOC111045711 (Nilaparvata lugens)
IIV6-SBPH-2	TCONS_00002613	XP_015509342	−	121	9.00E−34	15	133	157991	157875	Predicted RNA-directed DNA polymerase from mobile element jockey-like (Neodiprion lecontei)
IIV6-SBPH-3	TCONS_00003466	XP_022197601	−	62	1.00E-18	307	367	157958	157897	Uncharacterized protein LOC111054806 (Nilaparvata lugens)
IIV6-SBPH-4	TCONS_00008260	XP_022206744	−	57	6.00E−16	294	349	158024	157969	Endochitinase A-like isoform X1 (Nilaparvata lugens)
IIV6-SBPH-5	TCONS_00012920	XP_022204073	−	72	7.00E−21	373	443	157976	157905	Uncharacterized protein LOC111060713 isoform X1 (Nilaparvata lugens)
IIV6-SBPH-6	TCONS_00014496	No blast hits	+	102	1.00E−31	2699	2799	157898	157998	No blast hits
IIV6-SBPH-7	TCONS_00014495					2617	2717			
IIV6-SBPH-8	TCONS_00015277	XP_014260631	+	89	6.00E−29	44	130	157872	157960	Uncharacterized protein LOC106673143 isoform X2 (Cimex lectularius)
IIV6-SBPH-9	TCONS_00016989	XP_022204984	−	84	1.00E−26	783	865	157953	157872	Armadillo segment polarity protein isoform X3 (Nilaparvata lugens)
IIV6-SBPH-10	TCONS_00018813	XP_014247467	+	96	8.00E−28	2929	3023	157865	157958	Cylicin-1 (Cimex lectularius)
IIV6-SBPH-11	TCONS_00020430	No blast hits	+	110	2.00E−33	64	171	157875	157984	No blast hits
IIV6-SBPH-12	TCONS_00020698	XP_022186703	−	120	9.00E−33	2471	2587	157989	157871	Sialin-like (Nilaparvata lugens)
IIV6-SBPH-13	TCONS_00022745	XP_022187702	−	114	3.00E−37	127	239	157987	157876	Tetratricopeptide repeat protein 39B-like (Nilaparvata lugens)
IIV6-SBPH-14	TCONS_00024976	XP_022185269	−	120	1.00E−34	1796	1913	157990	157872	Probable serine/threonine-protein kinase PBL3 (Nilaparvata lugens)
IIV6-SBPH-15	TCONS_00024975	XP_022185272	−	120	1.00E−34	1802	1919	157990	157872	Inhibitor of Bruton tyrosine kinase isoform X2 (Nilaparvata lugens)
IIV6-SBPH-16	TCONS_00025666	XP_022192571	+	239	8.00E−63	2053	2286	157871	158106	Homeobox protein Nkx-2.4-like (Nilaparvata lugens)
IIV6-SBPH-17	TCONS_00026424	XM_022334380	−	253	1.00E−64	3808	4055	158119	157872	Predicted Nilaparvata lugens coronin-2B-like (LOC111048487)
IIV6-SBPH-18	TCONS_00026423					3715	3962			
IIV6-SBPH-19	TCONS_00026425					3541	3788			

a List of SBPH transcripts that mapped to IIV-6 genome.

b ID of assembled SBPH transcript.

c GenBank accession number for annotated SBPH transcript.

d Annotations of assembled SBPH transcript.

**TABLE 3:** RPSlS-containing transcripts identified in assembled N. lugens (BPH) transcriptome

ID^*a*^	Transcriptome assembly ID^*b*^	GenBank accession no.^*c*^	Orientation	Length (nt)	E value	Match coordinate				Annotation^*d*^
						mRNA position		IIV-6 genome position		
						Start	End	Start	End	
IIV6-BPH-1	TCONS_00027247	XM_022348255	−	93	7E−32	1845	1936	157970	157878	Nilaparvata lugens UPF0046 protein C25E10.12-like (LOC111060582)
IIV6-BPH-2	TCONS_00024158	XM_022345494	+	109	1.00E−22	2109	2214	157918	158024	Nilaparvata lugens neuropeptide-like 1 (LOC111058001)
IIV6-BPH-3	TCONS_00030689	XM_022351452	+	201	3.00E−50	829	1023	157871	158069	Nilaparvata lugens protein-L-isoaspartate(d-aspartate) O-methyltransferase (LOC111063773)
IIV6-BPH-4	TCONS_00022544	XM_022344131	−	86	1.00E−29	1526	1610	157961	157876	Nilaparvata lugens N(4)-(Beta-N-acetylglucosaminyl)-l-asparaginase-like (LOC111056738)
IIV6-BPH-5	TCONS_00017127	XM_022339397	−	114	5.00E−29	1734	1843	158007	157895	Nilaparvata lugens sorting nexin-16-like (LOC111052656)
IIV6-BPH-6	TCONS_00016887	XM_022339164	+	120	4.00E−39	6461	6579	157872	157989	Nilaparvata lugens dedicator of cytokinesis protein 1 (LOC111052477) (isoform X1-X4)
	TCONS_00016886	XM_022339165				6476	6594			
	TCONS_00016888	XM_022339166				6479	6597			
	TCONS_00016885	XM_022339168				6572	6690			
IIV6-BPH-7	TCONS_00015794	XM_022338223	+	148	4.00E−45	2255	2401	157878	158023	Nilaparvata lugens U2 small nuclear ribonucleoprotein A′-like (LOC111051677)
IIV6-BPH-8	TCONS_00014979	XM_022337511	−	96	1.00E−24	3710	3804	157966	157871	Nilaparvata lugens zinc finger protein 208-like (LOC111051081)
IIV6-BPH-9	TCONS_00014261	XM_022336895	−	147	3.00E−48	2243	2388	158021	157875	Nilaparvata lugens nucleotide exchange factor SIL1 (LOC111050554) (isoform X1-X2)
	TCONS_00014262	XM_022336896				2260	2405			
IIV6-BPH-10	TCONS_00013374	XM_022336116	−	154	7.00E−37	7674	7826	158027	157875	Nilaparvata lugens uncharacterized LOC111049921 (LOC111049921)
IIV6-BPH-11	TCONS_00011505	XM_022334458	−	172	3.00E−55	1470	1638	158045	157875	Nilaparvata lugens thyroid transcription factor 1-like (LOC111048546)
IIV6-BPH-12	TCONS_00010901	XM_022333940	+	81	8.00E−20	154	231	157869	157949	Nilaparvata lugens phospholipid phosphatase 2-like (LOC111048093)
IIV6-BPH-13	TCONS_00007252	XM_022330812	−	149	2.00E−36	3049	3195	158018	157873	Nilaparvata lugens alpha-catulin (LOC111045409), transcript variant X2
IIV6-BPH-14	TCONS_00006635	XM_022330266	+	176	1.00E−63	4098	4270	157871	158045	Nilaparvata lugens zinc finger protein 708-like (LOC111044981)
IIV6-BPH-15	TCONS_00006097	XM_022329798	−	173	1.00E−62	1027	1196	158045	157874	Nilaparvata lugens electron transfer flavoprotein regulatory factor 1 (LOC111044608)
	TCONS_00006098	XM_022329797				1105	1274			
	TCONS_00006099	XM_022329795				1144	1313			
IIV6-BPH-16	TCONS_00003375	XM_022351811	−	129	1.00E−41	589	714	157999	157871	Nilaparvata lugens uncharacterized LOC111064129 (LOC111064129)
IIV6-BPH-17	TCONS_00002425	XM_022344945	−	52	6.00E−16	1401	1452	157988	157938	Nilaparvata lugens methionine aminopeptidase 1D, mitochondrial-like (LOC111057488)
	TCONS_00002428	XM_022344939				1304	1355			
	TCONS_00002427	XM_022344935				1405	1456			
	TCONS_00002426	XM_022344928				1355	1406			
IIV6-BPH-18	TCONS_00003674	No blast hits	+	95	8.00E−37	133	227	157906	157999	No blast hits
IIV6-BPH-19	TCONS_00009246	No blast hits	−	63	2.00E−15	952	1013	157935	157873	No blast hits
IIV6-BPH-20	TCONS_00012337	No blast hits	−	175	2.00E−63	150	320	158045	157873	No blast hits
IIV6-BPH-21	TCONS_00013433	XP_022191867	−	74	8.00E−27	1621	1694	158026	157953	Peptidyl-alpha-hydroxyglycine alpha-amidating lyase 1-like (Nilaparvata lugens)
IIV6-BPH-22	TCONS_00024640	XP_021938737	+	120	1.00E−36	722	838	157925	158043	Tubulin-specific chaperone cofactor E-like protein (Zootermopsis nevadensis)
IIV6-BPH-23	TCONS_00025168	No blast hits	+	156	4.00E−54	305	459	157874	158028	No blast hits
IIV6-BPH-24	TCONS_00026803	No blast hits	+	124	7.00E−31	289	410	157926	158042	No blast hits

a List of BPH transcripts that mapped to IIV-6 genome.

b ID of assembled BPH transcript.

c GenBank accession number for annotated BPH transcript.

d Annotations of assembled BPH transcript.

Notably, none of the RPSlSs were integrated into the coding regions of predicted planthopper genes (Fig. 2). This may be because the disruption of the coding genes leads to detrimental effects on the insects. A previous study showed that transposable elements in the genome can be expressed at low levels and can play important roles in the regulation of gene expression (40, 41). Whether the RPSlSs inserted into planthopper genomes have similar transposon-like functions as the regulators of gene expression in rice planthoppers needs further investigation.

To investigate the expression profile of RPSlS loci at different planthopper developmental stages, seven RPSlSs of N. lugens were selected for RT-quantitative PCR (qPCR) analysis. There were relatively low expression levels in eggs or first-instar nymphs (except transcript TCONS_00024158) and markedly high expression in late-instar nymphs and adults (Fig. 3B). This result shows that RPSlSs containing transcriptions are differently regulated during the different developmental stages of N. lugens.

### Characteristics of RPSlS-derived small RNAs.

The canonical function of the piRNA pathway is in defense against transposable elements and to protect the integrity of the genome in both germ line and gonadal somatic cells of animal species (42). Recent results in mosquitos suggest that piRNAs can also be produced by endogenous flaviviral elements and play a role in insect antiviral immunity (12, 38). Thus, it is interesting to investigate whether RPSlS loci produce small RNAs. Nine publicly available small RNA libraries of three planthoppers were mapped to the complete genome of IIV-6 (NC_003038.1). Of the small RNA reads that mapped to the IIV-6 genome, 70.5% to 93.2% of the unique small RNA reads and 64.8% to 96.8% of redundant reads mapped to the IIV6_300 sequence, which indicates the accumulation of small RNAs derived from RPSlS loci (Table 4).

**TABLE 4:** Numbers of reads of small RNAs of three planthoppers mapped to IIV-6 genome (allowing 1 mismatch)

Species and small RNA libraries^*a*^ mapped to IIV-6 genome	Unique reads		Redundant reads	
	Mapped to IIV-6 genome (total no.)	Mapped to IIV6_300 [no. (%)]	Mapped to IIV-6 genome (total no.)	Mapped to IIV6_300 [no. (%)]
L. striatellus				
LS_VF	89	68 (76.4)	105	76 (72.4)
LS_RB	72	59 (81.9)	86	71 (82.6)
LS_RSV	71	61 (85.9)	101	91 (90.1)
LS_DI	60	49 (81.7)	69	58 (84.1)
N. lugens				
NL_CC	133	118 (88.7)	193	177 (91.7)
NL_CX	105	74 (70.5)	162	105 (64.8)
NL_CY	71	61 (85.9)	94	81 (86.2)
S. furcifera				
SF_VF	924	861 (93.2)	2,951	2,857 (96.8)
SF_SRB	823	766 (93.1)	2,694	2,592 (96.2)

a LS_VF, virus-free adults of L. striatellus; LS_RB, adults of L. striatellus infected with RBSDV; LS_RSV, adults of L. striatellus infected with RSV; LS_DI, adults of L. striatellus with mixed infection of RBSDV and RSV; NL_CC, female adults of N. lugens; NL_CX, male adults of N. lugens; NL_CY, last-instar female nymph of N. lugens; SF_VF, virus-free adults of S. furcifera; SF_SRB, adults of S. furcifera infected with SRBSDV.

**FIG 4:**
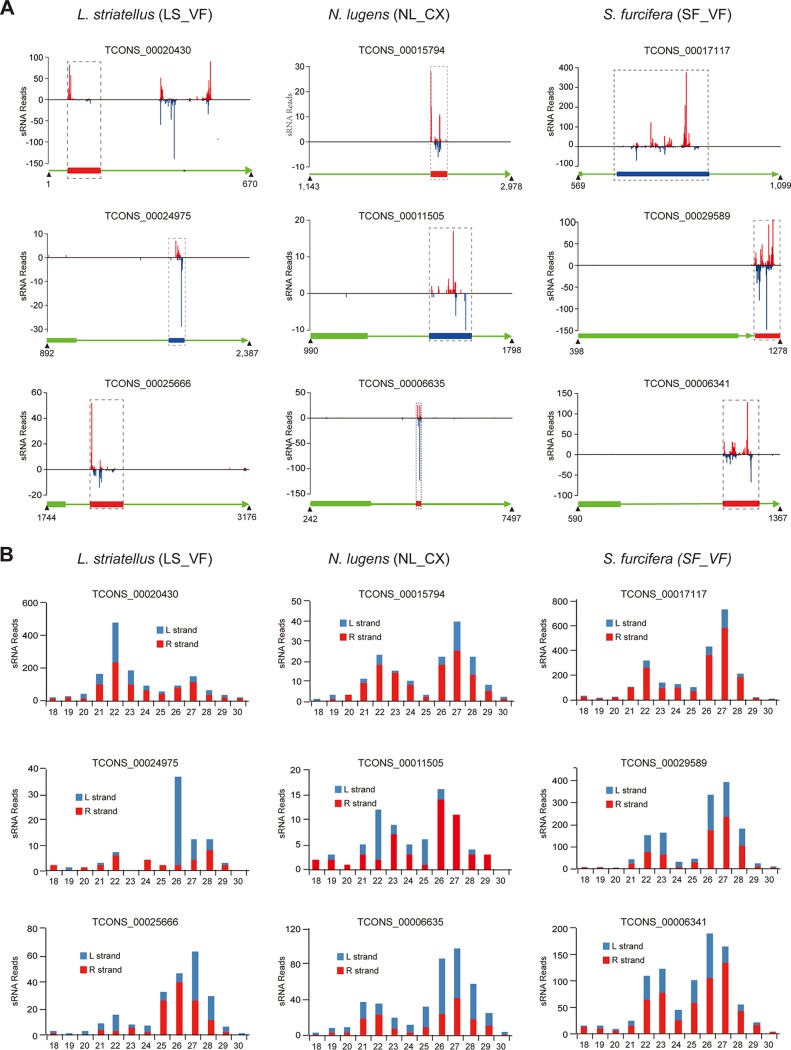
Production of small RNAs derived from RPSlS loci in planthoppers. (A) Mapping of small RNAs (18 to 30 nt) to the planthopper transcripts containing RPSlSs. Red and blue colors indicate small RNAs derived from the sense and antisense strands, respectively, of planthopper transcripts. Schematic representation below each plot shows the organization of the transcripts and the position of small RNAs in the transcripts. Green lines with arrows indicate planthopper transcripts. Green boxes represent the predicted ORF within insect transcripts. Red and blue boxes represent the transcripts of RPSlSs with R and L strands, respectively. One mismatch was allowed during small RNA mapping. (B) Size distribution of small RNAs derived from the RPSlS loci mapped to planthopper transcripts, corresponding to the mapping shown in panel A. LS_VF, virus-free adults of L. striatellus. NL_CX, male adults of N. lugens. SF_VF, virus-free adults of S. furcifera.

**FIG 5:**
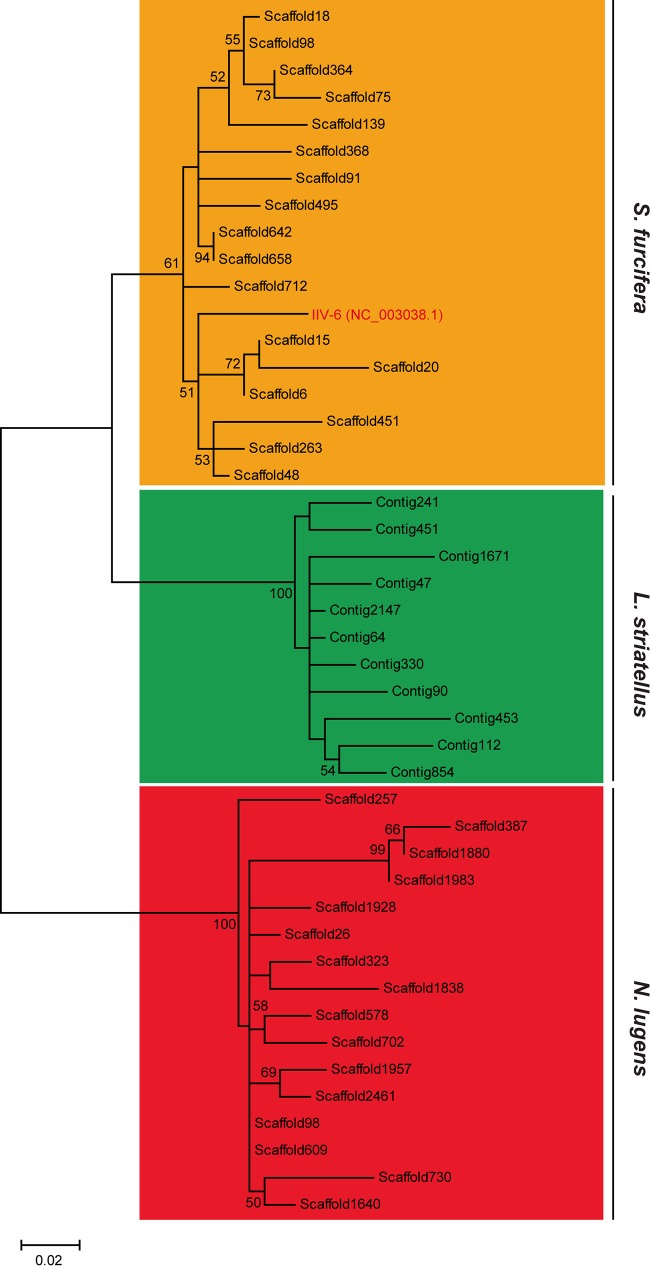
Phylogenetic analysis of RPSlSs (L strand) in three planthoppers using the maximum likelihood algorithm. Numbers at each branch node represent the values calculated by bootstrap analysis (1,000 replications; only values of >50 are shown). Exogenous IIV-6 (IIV6_300, with the corresponding range and orientation) is indicated with red font.

More RPSlS-derived small RNAs were identified in S. furcifera than in the other two planthoppers (Table 4), perhaps because of the closer relationship of RPSlSs in S. furcifera with the reference exogenous IIV-6 (see Fig. 5). Since there are some sequence variations among RPSlSs from the three rice planthoppers and exogenous IIV-6, and for a better understanding of the production of RPSlS-derived small RNAs, small RNA libraries of LS_VF (L. striatellus), NL_CX (N. lugens), and SF_VF (S. furcifera) were further mapped to three randomly selected RPSlS-containing transcripts from corresponding planthoppers. As expected, more small RNAs derived from RPSlS loci were identified by this method (Table 4). Evidently, small RNAs were specifically mapped to RPSlS regions except for the TCONS_00020430 transcript (Fig. 4A). Obvious small RNA hotspots were observed, and these were usually identified in both strands (Fig. 4A). Interestingly, RPSlS-derived small RNAs are predominantly 26 to 28 nt, followed by a 21- to 23-nt peak, although TCONS_00020430 has a clear 22-nt peak (Fig. 4B). However, a 26- to 28-nt small RNA peak was observed in TCONS_00020430 if only the RPSlS region of the transcript was mapped (data not shown), suggesting that the abundant small RNAs with a length of 21 to 23 nt are mainly derived from different regions of the transcript.

The production of piRNAs (a class of the small RNAs) from endogenous viral elements was recently reported from mosquitos; these were antisense strand and could target cognate viral RNA (11, 12, 38). Previous studies indicated that the piRNA pathway plays an essential role in antiviral defense of mosquitos but not of other insects, such as a fly (*Drosophila* spp.) (43). Another study demonstrated that exogenous IIV-6, as a dsDNA virus, triggers an RNA interference-based antiviral defense mechanism in *Drosophila* with the generation of virus-derived small interfering RNA in a DICER2 (RNase III enzyme)-dependent manner (44). From our results, small RNAs derived from RPSlS loci were predominantly 26 to 28 nt long, which is a typical characteristic of piRNAs (24 to 32 nt) (45). We therefore extracted RPSlS-derived small RNAs with lengths of 26 to 28 nt for further sequence logo analysis (https://weblogo.berkeley.edu/logo.cgi). However, this analysis did not identify another typical characteristic of piRNAs, namely, a strong U bias at the 5′ terminus or enrichment of A at nt 10 (42 and data not shown). It is remains unclear whether RPSlS-derived small RNAs function in the piRNA pathway against transposons. It will also be interesting to further investigate whether RPSlS-derived small RNAs could mediate antiviral defense against IIV-6 infection.

### Phylogenetic relationship of IIV6_300 and RPSlSs.

A phylogenetic tree was constructed based on the RPSlSs using the maximum likelihood method. Evidently, RPSlSs were grouped according to the insect species with strong bootstrap support (Fig. 5). IIV6_300 sequence is clustered with RPSlSs of S. furcifera, indicating that IIV-6 obtained a SINE-like transposable element from S. furcifera in the past after the evolutionary divergence of the three rice planthoppers. Note that we could not find any homologous sequence to RPSlSs in other viruses deposited in the public database. Considering that IIV-6 is a giant DNA virus that commonly obtains genetic material from the host, it is very likely that the transposable element is transferred from a planthopper host to the IIV-6 genome. It will be interesting to investigate the possible HT of RPSlSs between eukaryotic organisms involving virus vectors, as recently reported for other viruses and hosts (14–16).

In conclusion, our investigation on possible occurrences of HT between rice planthoppers and viruses leads to the finding of newly identified retroposon-like elements that transfer to an iridovirus. To the best of our knowledge, this is the first report of a potential HT event between a planthopper and a giant DNA virus and also the first evidence for the eukaryotic origin of genetic material in iridoviruses. The results of this study will further contribute to our understanding of HT events between viruses and their eukaryotic hosts.

## MATERIALS AND METHODS

### Insect cultures.

Populations of three planthoppers (L. striatellus, N. lugens, and S. furcifera) that were not carrying the known rice viruses were reared on susceptible rice seedlings (cv. Wuyujing no. 3) in climate-controlled rooms at 26°C ± 1°C, with a photoperiod of 16 h of light and 8 h of darkness and 70% ± 10% relative humidity.

### VLSs in three rice planthopper genomes.

The assembled genomes of L. striatellus, N. lugens, and S. furcifera were retrieved from Gigadb and the NCBI reference genome database (33–35). These genomes were searched against NCBI virus RefSeqs (ftp://ftp.ncbi.nlm.nih.gov/refseq/release/viral) using a BLASTN algorithm with a cutoff E value of ≤10^−5^. The detected virus-like sequences (VLSs) are listed in File S1 in the supplemental material. Since most of the planthopper VLSs (>90%) were mapped to a restricted region (∼300 nt) of IIV-6 (IIV6_300), the three planthopper genomes were then searched directly against the IIV-6 genome (NC_003038.1) to identify the IIV-6-like nucleotide sequences (sequences homologous to IIV6_300) in planthoppers. BLAST results are listed in File S2. In addition, contig/scaffold regions of planthoppers that mapped to IIV-6 were further extracted and extended 500 bases at both 5′ and 3′ termini (to the end of the termini) and used for the identification of potential transposable elements with CENSOR (https://www.girinst.org/censor/index.php).

### IIV6_300-like sequences containing transcripts identified from reassembled rice planthopper transcriptomes.

Transcriptome raw data were downloaded from the NCBI Sequence Read Archive (SRA) database for L. striatellus (SRX2013762), N. lugens (SRX023419), and S. furcifera (SRX104935). The filtered transcriptome raw reads were then aligned against their corresponding genomes using Tophat2 (http://ccb.jhu.edu/software/tophat/index.shtml) and reassembled using Cufflinks (http://cole-trapnell-lab.github.io/cufflinks/). The newly reassembled transcripts of the three planthoppers are available upon request. The assembled transcriptomes were also searched again against the IIV-6 genome (NC_003038.1) using BLASTN (E value of ≤10^−5^) to identify the transcripts containing the IIV6_300-like sequence (rice planthopper SINE-like sequences, or RPSlSs). The identified planthopper transcripts were then searched against NCBI NR (NCBI nonredundant protein sequences) and NT (nucleotide sequences) databases for annotation. The results are listed in Table 2 (L. striatellus), Table 3 (N. lugens), and Table S1 (S. furcifera). Furthermore, to determine the accurate location of the RPSlS within the planthopper transcripts and genome, the planthopper transcripts containing RPSlSs were used as a query to search against the genome of the three planthoppers using BLASTN (E value of ≤10^−10^), and the results are available upon request.

### Detection of planthopper scaffolds/contigs containing RPSlSs.

Genomic DNAs were extracted from the three planthoppers using an insect DNA extraction kit (Omega, USA) following the manufacturer’s instructions. Five scaffold/contig sequences (partial, ∼500 to ∼700 bp, containing RPSISs) from each planthopper were randomly selected to verify the presence of RPSISs. The PCR products of each sample were purified, ligated into the pMD18-T vector (TaKaRa, China), and sequenced (Tsingke, China). The primer sets used for genome amplification are listed in Table 5.

**TABLE 5:** Primer sets used in this study

Primer name	Primer sequence (5′–3′)
Ls-DNA-Contig8-1	F, TCAATTGATGCTCAATCAACTTCC; R, TGGGTTTTCATTAATAGAGCGAGT
Ls-DNA-Contig1-2	F, ACTCCAATTGTCTCTGCTTACA; R, TCATATTTGGTGAAGTCTCCTCA
Ls-DNA-Contig157-1	F, GTTAGTTGCCAACCAGCCTA; R, GTGATAACGGTCTTTCCCCG
Ls-DNA-Contig0-1	F, CGAAGCTGTTGCACACAATC; R, CGTTACTGGTACTTTCCCAGA
Ls-DNA-Contig30-1	F, GAGGTATCGCGCTACTCTTTTT; R, TCATGGTATCTGCCCTGCCT
Nl-DNA-Scaffold4554-1	F, GTGATGAGTGGAAGAAGGTGA; R, CGTTCATACACTCTTACCCGA
Nl-DNA-Scaffold2050-1	F, AAGCTAAGCGTAATTTGGGC; R, CCTCTACATTTATCAGGAAATACGC
Nl-DNA-Scaffold6-12	F, TACCGGTATCAGCAGTCATCT; R, GACTTGTTCTGGCCTTGTCG
Nl-DNA-Scaffold727-2	F, GATTGATGTGTCCATTTTCGGG; R, GAACCTGAGCAGAGTAAGTCG
Nl-DNA-Scaffold1287-11	F, GGGAAACGTAAAATCGGCGT; R, ATTTTGAGTTTAAGCCACCAGC
Sf-DNA-Scaffold20-60	F, CCACTGGCGGTGGAATTATTTTAT; R, CAGTAGGCTGTTTGTGTTTCAT
Sf-DNA-Scaffold24-9	F, TGCCTCGATCTGGAAGTACA; R, GCCTGTTAAGCTAACTTTGTGG
Sf-DNA-Scaffold8-1	F, GATTCTTGTGAGCCCAGTGAG; R, CTTCACAAGTGAGCTTTAAGGGG
Sf-DNA-Scaffold15-1	F, CTTCTGGGGAAAACTGGAGC; R, TGTTAAATTGATGTGGAAAGCAAA
Sf-DNA-Scaffold17-1	F, ACATCATTCTGGCACTCTTTTTCA; R, AAATTATTCCCCCTGACATTCATTT
Ls-Transcripts-TCONS_00026424	F, GAATATGTGTCTGGCATTCCTCA; R, CCAAGCGCTCGTCACTTATC
Ls-Transcripts-TCONS_00008260	F, ACAGAAAGCAACTGAGGTGTAAC; R, ACCTGAGCCTTTGGCTTGTG
Ls-Transcripts-TCONS_00002613	F, TGCTTGAGATAATCCGGCTG; R, TCAAGCCTGATGTTTGATGGG
Ls-Transcripts-TCONS_00025666	F, ACCCTCATCGTCACTCACATC; R, GCGCATGCGTCAACGAAAAA
Ls-Transcripts-TCONS_00003466	F, TCCTCTGGTAGGAGGTTGCC; R, AGGAACACCTGAAGCATCAAC
Nl-Transcripts-TCONS_00024158	F, CGACAAATCGTGTAGTCGCT; R, TCTTCGACTCAATTTTCGGGA
Nl-Transcripts-TCONS_00022544	F, CTACAATGTTATTATAGGAGCCGTG; R, TTTTCTCTGGCTCAGTCTCTTAATC
Nl-Transcripts-TCONS_00017127	F, ACTGGAAAGTTTTGATACTGTTTCT; R, CAGACAACTGTGGCTGCTAT
Nl-Transcripts-TCONS_00014979	F, TGTTGTAACTCATCAAACAGTGG; R, AAACCATTTATATCACAGATAGCCT
Nl-Transcripts-TCONS_00006635	F, CCCACATTTGAAAGTGATCATAGC; R, AAGAACAACGACAACAATTATGGAT
Sf-Transcripts-TCONS_00023659	F, GACCGACGGCTTAACGTGT; R, CCGTTCGAGAGTGACAGCAG
Sf-Transcripts-TCONS_00026590	F, GGGGATCTCGAAACCGTCCA; R, TACTCCAGCTCGGTGAATATTGG
Sf-Transcripts-TCONS_00031118	F, TGAGCGTGCTCTGACATGGA; R, GACTTTGGTTTTTCGGCGCTT
Sf-Transcripts-TCONS_00036274	F, TACAGCGGTTGTGGTCCGT; R, AAGCCGGCCAAGTCGGA
Sf-Transcripts-TCONS_00006341	F, TGTCAGGTTTACCGTTCAGAC; R, AGGCATACTCCAGAGATAACCAA
qPCR-Nl-18S	F, GTAACCCGCTGAACCTCC; R, GTCCGAAGACCTCACTAAATCA
qPCR-Nl-TCONS_00024158	F, ATAATAATATTGGGTGACATGGCTG; R, TGAGTCTCTATCGATTTTCTTGTTG
qPCR-Nl-TCONS_00022544	F, CAATGTTATTATAGGAGCCGTGAGT; R, TGTCAGAGTTTTCAGGTCGCA
qPCR-Nl-TCONS_00017127	F, CCCGACTGCCTGAAAAACAG; R, GTTATCAGACAACTGTGGCTGC
qPCR-Nl-TCONS_00016885	F, TGGGTTGATTCATCTTCGAGTT; R, CGCCAAGGCTGCCTAAAAAG
qPCR-Nl-TCONS_00014979	F, AAGCTATCGCGTTTGTAAAGCTG; R, TTTGCCAAGCTGTGAACACTC
qPCR-Nl-TCONS_00014262	F, TGCTTCCATTCCATTCAAGCC; R, TTGCTGCGTCCAATTTGTGG
qPCR-Nl-TCONS_00006635	F, GGCGACGTTGGCACATTAC; R, ATGGACACGTTAAGCCGTCG

### Detection of planthopper transcripts containing RPSIS.

Total RNAs were extracted from the three planthoppers using TRIzol reagent (Invitrogen, USA). The purified RNAs were mixed with genomic DNA remover (Toyobo, Japan) and used for RT-PCR. cDNA was synthesized using HiScript II reverse transcription (Vazyme, China) according to the manufacturer’s instructions. Five partial transcripts (approximately 500 bases) containing RPSISs from each planthopper were randomly selected to confirm the expression of RPSISs. The PCR products of each sample were also sequenced as described above. The positions of the primer sets used to amplify the transcripts are shown by red arrows in Fig. 2, and the primer sequences are listed in Table 5.

### Expression analysis of RPSISs containing RNAs in N. lugens.

To determine the expression of RPSISs containing transcripts in N. lugens at different developmental stages, samples from eggs, first-instar nymphs, second- and third-instar nymphs, fourth- and fifth-instar nymphs, and female and male adults were collected for RNA extraction. Equal quantities of total RNA from each sample were used for cDNA synthesis, as described earlier. Primer sets specific for the seven transcripts containing RPSIS were used for RT-qPCR using the 18S rRNA of N. lugens as an internal reference gene. The primer sequences are listed in Table 5. Three independent biological replicates were used in this experiment.

### Small RNA analysis derived from RPSIS loci.

To investigate the possible presence of small RNAs derived from RPSIS loci, nine publicly available small RNA libraries of three rice planthoppers were retrieved. Four L. striatellus libraries were downloaded from the NCBI SRA database: LS_VF (virus-free adults, SRA no. SRX255768), LS_RB (adults infected with RBSDV, SRA no. SRX255770), LS_RSV (adults infected with RSV, SRA no. SRX255771), and LS_DI (adults with mixed infections of RBSDV and RSV, SRA no. SRX255769). Three N. lugens libraries were kindly provided by Yongjun Lin, Huazhong Agricultural University (46): NL_CC (female adults), NL_CX (male adults), and NL_CY (last-instar female nymph). Two S. furcifera libraries were downloaded from the NCBI SRA database: SF_VF (virus-free adults, SRA no. SRX1544811) and SF_SRB (adults infected with SRBSDV, SRA no. SRX1546399). These 9 small RNA libraries were first mapped to the genome of IIV-6 (NC_003038.1), and then 3 small RNA libraries (LS_VF, NL_CX, and SF_VF) were further mapped to three randomly selected transcripts containing RPSISs (>100 bases) from each planthopper.

For small RNA bioinformatics analysis, preliminary treatment of the raw data was performed as described previously (47). In brief, small RNAs with lengths of 18 to 30 nt were extracted and collapsed for downstream analysis after 3′ adaptor removal and treatment of low-quality and junk sequences. The treated small RNAs of each library were mapped to the IIV-6 genome (NC_003038.1) using Bowtie software (http://bowtie-bio.sourceforge.net/index.shtml), allowing for one mismatch to identify RPSIS-derived small RNAs. In addition, to confirm the presence of RPSIS small RNA within planthopper transcripts, three planthopper small RNA libraries (LS_VF, NL_CX, and SF_VF) were mapped to three randomly selected transcripts (containing RPSISs) from each planthopper. The subsequent analyses were performed using custom Perl scripts and Linux bash scripts.

### Phylogenetic analysis of RPSISs.

Relatively long RPSISs (the L strand) from each planthopper were selected and aligned in ClustalW implemented in MEGA (version 6) (48), followed by manual editing. Planthopper sequences mapped to a region from nt 158120 to 157874 of the IIV-6 genome (the region of ORFs 353L and 354L of IIV6_300) were used for phylogenetic analysis considering the length concordant to the aligned RPSISs. The only one exogenous IIV-6 (IIV6_300) with the corresponding range and orientation available at present was included in this analysis. Phylogenetic analysis was carried out using MEGA 6, and the tree was generated using the maximum likelihood algorithm (1,000 bootstrap replications) (48).

## Supplementary Material

Supplemental file 1

Supplemental file 2

Supplemental file 3
