# Supplementary material for: Horizontal Transfer of a Retrotransposon from the Rice Planthopper to the Genome of an Insect DNA Virus
Source: J Virol. 2019 Mar 5;93(6):e01516-18. doi: 10.1128/JVI.01516-18 (PMC6401454; doi:10.1128/JVI.01516-18)
Supplement: Supplemental file 3 [file JVI.01516-18-s0003.pdf]

Table S1. RPSlS-containing transcripts identified in assembled *S. furcifera* (WBPH) transcriptome

ID[1]	Transcriptome Assembly ID[2]	GenBank accession no.[3]	Orientation	Length	E-value	Match coordinate				Annotation[4]
						mRNA position		IIV-6 genome position		
						Start	End	Start	End	
IIV6-WBPH-1	TCONS_00000821	XP_022197877	-	95	6.00E-35	1	94	157963	157870	uncharacterized protein CG45076-like isoform X2 [*Nilaparvata lugens*]
IIV6-WBPH-2	TCONS_00000826				4.00E-35					
IIV6-WBPH-3	TCONS_00000822	XP_022203711	-	95	6.00E-35	1	94	157963	157870	caldesmon-like, partial [*Nilaparvata lugens*]
IIV6-WBPH-4	TCONS_00000815				2.00E-34					
IIV6-WBPH-5	TCONS_00001403	XP_022183852	+	68	3.00E-25	1376	1443	157982	158049	uncharacterized protein LOC111043255 [*Nilaparvata lugens*]
IIV6-WBPH-6	TCONS_00001400	XP_022197966	+	68	4.00E-25	1881	1948	157982	158049	zinc transporter ZIP1 [*Nilaparvata lugens*]
IIV6-WBPH-7	TCONS_00001401			68		2083	2150			
IIV6-WBPH-8	TCONS_00001615	BAO01081	+	104	1.00E-43	501	604	157982	158085	neuropeptide GPCR A31 [*Nilaparvata lugens*]
IIV6-WBPH-9	TCONS_00001675	XP_022204966	-	69	3.00E-28	1	69	157963	157895	cilia- and flagella-associated protein 57-like [*Nilaparvata lugens*]
IIV6-WBPH-10	TCONS_00001799	XP_022199973	-	198	1.00E-69	969	1164	158085	157896	adenylyl cyclase-associated protein 1 isoform X1 [*Nilaparvata lugens*]
IIV6-WBPH-11	TCONS_00001798					1094	1289			
IIV6-WBPH-12	TCONS_00002092	XP_022189018	-	82	5.00E-35	1	82	157963	157882	calpain-A isoform X3 [*Nilaparvata lugens*]
IIV6-WBPH-13	TCONS_00002163	XP_022200385	-	68	2.00E-29	1	68	157963	157896	uncharacterized protein LOC111057264 [*Nilaparvata lugens*]
IIV6-WBPH-14	TCONS_00002498	XP_022192543	-	102	6.00E-28	701	794	157997	157897	tyrosine-protein phosphatase non-receptor type 61F-like [*Nilaparvata*

IIV6-WBPH-15	TCONS_00002497									*lugens*]
IIV6-WBPH-16	TCONS_00002741	XP_022203703	-	154	1.00E-71	1	154	158050	157897	uncharacterized protein LOC111060349 isoform X1 [*Nilaparvata lugens*]
IIV6-WBPH-17	TCONS_00002740	XP_022197379	-	67	7.00E-28	1	67	157963	157897	DNA-binding protein D-ETS-4 isoform X2 [*Nilaparvata lugens*]
IIV6-WBPH-18	TCONS_00003136	XP_022198750	-	63	5.00E-20	9294	9356	158122	158060	katanin p60 ATPase-containing subunit A-like 1 [*Nilaparvata lugens*]
IIV6-WBPH-19	TCONS_00004025	No blast hits	-	92	2.00E-33	1	91	157963	157872	No blast hits
IIV6-WBPH-20	TCONS_00004198	XP_022194996	-	118	3.00E-46	68	185	158014	157897	proton-coupled folate transporter-like isoform X3 [*Nilaparvata lugens*]
IIV6-WBPH-21	TCONS_00004199	No blast hits	-	118	1.00E-46	68	185	158014	157897	No blast hits
IIV6-WBPH-22	TCONS_00004538	XP_022189797	-	69	9.00E-28	1	69	157963	157895	paired box pox-neuro protein [*Nilaparvata lugens*]
IIV6-WBPH-23	TCONS_00004656	XP_022207153	+	183	3.00E-84	1463	1645	157870	158052	heparan-alpha-glucosaminide N-acetyltransferase isoform X1 [*Nilaparvata lugens*]
IIV6-WBPH-24	TCONS_00004657					1521	1703			
IIV6-WBPH-25	TCONS_00004812	XP_022192388	+	80	3.00E-20	396	474	157988	158067	putative leucine-rich repeat-containing protein DDB_G0290503 [*Nilaparvata lugens*]
IIV6-WBPH-26	TCONS_00005413	XP_022202387	-	139	5.00E-60	485	623	158034	157896	CXXC-type zinc finger protein 1-like [*Nilaparvata lugens*]
IIV6-WBPH-27	TCONS_00005976	AHB59663	-	192	1.00E-82	465	656	158087	157896	odorant-binding protein 6 [*Sogatella furcifera*]
IIV6-WBPH-28	TCONS_00006415	No blast hits	+	168	2.00E-77	929	1096	157877	158044	No blast hits
IIV6-WBPH-29	TCONS_00006341	XP_022202296	-	143	2.00E-61	1152	1294	158010	157870	pre-mRNA-splicing factor syf2 [*Nilaparvata lugens*]
IIV6-WBPH-30	TCONS_00006633	No blast hits	+	143	7.00E-54	890	1032	157984	158126	No blast hits

IIV6-WBPH-31	TCONS_00006773	XP_022186303	+	128	5.00E-39	1140	1265	157873	157998	glucose dehydrogenase [FAD, quinone]-like [*Nilaparvata lugens*]
IIV6-WBPH-32	TCONS_00006956	KYQ49512	-	128	1.00E-38	5460	5585	157998	157873	Glucose dehydrogenase [acceptor] [*Trachymyrmex zeteki*]
IIV6-WBPH-33	TCONS_00008012	XP_022181088	-	61	5.00E-23	166	226	157956	157896	uncharacterized protein K02A2.6-like, partial [*Myzus persicae*]
IIV6-WBPH-34	TCONS_00008489	No blast hits	-	122	9.00E-48	179	300	158123	158002	No blast hits
IIV6-WBPH-35	TCONS_00008663	XP_022202371	-	89	8.00E-34	918	1006	157964	157876	dual oxidase maturation factor 1-like [*Nilaparvata lugens*]
IIV6-WBPH-36	TCONS_00008665	No blast hits	-	88	1.00E-34	1	87	157963	157876	No blast hits
IIV6-WBPH-37	TCONS_00008951	No blast hits	-	86	1.00E-36	1	86	157956	157871	No blast hits
IIV6-WBPH-38	TCONS_00009589	No blast hits	+	145	7.00E-48	578	714	157871	158015	No blast hits
IIV6-WBPH-39	TCONS_00009929	No blast hits	-	89	2.00E-34	711	799	157958	157871	No blast hits
IIV6-WBPH-40	TCONS_00010071					967	1088			
IIV6-WBPH-41	TCONS_00010072	XP_022189254	-	123	4.00E-42	1135	1256	158123	158002	uncharacterized protein LOC111047734 [*Nilaparvata lugens*]
IIV6-WBPH-42	TCONS_00010070					1658	1779			
IIV6-WBPH-43	TCONS_00010587	XP_022190214	-	115	5.00E-47	3658	3771	158087	157973	SWI/SNF-related matrix-associated actin-dependent regulator of chromatin subfamily A containing DEAD/H box 1 homolog [*Nilaparvata lugens*]
IIV6-WBPH-44	TCONS_00011015	XP_022191123	+	102	6.00E-39	811	912	157983	158084	tyrosine 3-monooxygenase [*Nilaparvata lugens*]
IIV6-WBPH-45	TCONS_00011036	XP_022198364	-	67	2.00E-28	1	67	157963	157897	melanotransferrin [*Nilaparvata lugens*]
IIV6-WBPH-46	TCONS_00012703	XP_022200551	-	69	1.00E-24	1	69	157963	157895	homeobox protein engrailed-1-B-like [*Nilaparvata lugens*]

IIV6-WBPH-47	TCONS_00012460	XP_022204650	-	80	3.00E-29	915	994	157954	157876	uncharacterized protein LOC111061260 [*Nilaparvata lugens*]
IIV6-WBPH-48	TCONS_00012459					1171	1250			
IIV6-WBPH-49	TCONS_00012728	No blast hits	-	67	1.00E-28	1	67	157963	157897	No blast hits
IIV6-WBPH-50	TCONS_00013319				3.00E-60	330	471			
IIV6-WBPH-51	TCONS_00013318	No blast hits	+	142	7.00E-60	516	657	157982	158123	No blast hits
IIV6-WBPH-52	TCONS_00013317				8.00E-60	696	837			
IIV6-WBPH-53	TCONS_00013294	XP_018904403	-	128	2.00E-49	260	387	158023	157897	XP_018904403
IIV6-WBPH-54	TCONS_00013293				9.00E-50	260	387			
IIV6-WBPH-55	TCONS_00013807					7996	8072			
IIV6-WBPH-56	TCONS_00013809	XP_022185829	-	77	6.00E-33	7636	7712	157958	157882	uncharacterized protein LOC111044883 []
IIV6-WBPH-57	TCONS_00013808					7927	8003			
IIV6-WBPH-58	TCONS_00013806					7837	7913			
IIV6-WBPH-59	TCONS_00013970			94	1.00E-39	2481	2574	157966	157873	
IIV6-WBPH-60	TCONS_00013970	XP_022189171	-	61	1.00E-19	909	969	158120	158060	Kv channel-interacting protein 1 isoform X1 [*Nilaparvata lugens*]
IIV6-WBPH-61	TCONS_00013974			94	2.00E-39	2738	2831	157966	157873	
IIV6-WBPH-62	TCONS_00013974			61	2.00E-19	1166	1226	158120	158060	
IIV6-WBPH-63	TCONS_00014074	XP_023709721	-	103	1.00E-38	3124	3226	157966	157865	protein tincar [*Cryptotermes secundus*]

IIV6-WBPH-64	TCONS_00014500	XP_022192291	-	97	1.00E-37	1119	1214	158129	158033	gastrula zinc finger protein XlCGF67.1-like isoform X1 [*Nilaparvata lugens*]
IIV6-WBPH-65	TCONS_00014986	XP_022197639	+	101	9.00E-42	1751	1851	157881	157981	uncharacterized protein LOC111054836, partial [*Nilaparvata lugens*]
IIV6-WBPH-66	TCONS_00014960	XP_022200896	-	80	6.00E-36	1	80	157963	157884	probable medium-chain specific acyl-CoA dehydrogenase, mitochondrial [*Nilaparvata lugens*]
IIV6-WBPH-67	TCONS_00015041	XP_022191828	+	218	8.00E-79	3824	4040	157862	158077	G-protein-signaling modulator 2 [*Nilaparvata lugens*]
IIV6-WBPH-68	TCONS_00015042				8.00E-79	3932	4148			
IIV6-WBPH-69	TCONS_00015142		-	67	1.00E-27	1	67	157963	157897	
IIV6-WBPH-70	TCONS_00015141				7.00E-28	1	67			
IIV6-WBPH-71	TCONS_00015658	XP_022189872	-	67	9.00E-25	1	67	157963	157897	cyclin-dependent kinase 14-like [*Nilaparvata lugens*]
IIV6-WBPH-72	TCONS_00015883	XP_022190759	-	91	1.00E-35	192	279	157959	157869	uncharacterized protein LOC111049065 [*Nilaparvata lugen*s]
IIV6-WBPH-73	TCONS_00015884				6.00E-35					
IIV6-WBPH-74	TCONS_00015935	XP_022193003	+	141	2.00E-39	2297	2437	157871	158007	shootin-1-like [*Nilaparvata lugens*]
IIV6-WBPH-75	TCONS_00016148	XP_022186073	-	67	9.00E-27	1	67	157963	157897	protein BTG2-like [*Nilaparvata lugens*]
IIV6-WBPH-76	TCONS_00016227	No blast hits	-	141	7.00E-40	222	362	158007	157871	No blast hits
IIV6-WBPH-77	TCONS_00016228	XP_022193004	-	141	2.00E-39	2256	2396	158007	157871	inhibin beta C chain isoform X1 [*Nilaparvata lugens*]
IIV6-WBPH-78	TCONS_00016843	XP_022187258	-	131	6.00E-55	3165	3295	157995	157865	uncharacterized protein LOC111046076 [Nilaparvata lugens]
IIV6-WBPH-79	TCONS_00017117	XP_022188809	-	246	2.00E-104	671	916	158110	157865	uncharacterized protein LOC111047379, partial [*Nilaparvata*

										lugens]
IIV6-WBPH-80	TCONS_00017605	No blast hits	+	116	9.00E-46	473	588	157987	158102	No blast hits
IIV6-WBPH-81	TCONS_00017982	XP_022190218	-	90	1.00E-32	1072	1161	157966	157877	mpv17-like protein 2 [*Nilaparvata lugens*]
IIV6-WBPH-82	TCONS_00018542	PNF35738	+	136	2.00E-51	1471	1606	157983	158118	hypothetical protein B7P43_G15311 [*Cryptotermes secundus*]
IIV6-WBPH-83	TCONS_00019470	XP_022198019	-	140	2.00E-57	6851	6989	158034	157896	spectrin beta chain, partial [*Nilaparvata lugens*]
IIV6-WBPH-84	TCONS_00019585	XP_022196748	-	102	6.00E-40	3997	4098	158008	157908	glutaminase liver isoform, mitochondrial [*Nilaparvata lugens*]
IIV6-WBPH-85	TCONS_00019586		-	102	6.00E-40	4174	4275	158008	157908	
IIV6-WBPH-86	TCONS_00019766	XP_022202924	-	191	1.00E-57	1460	1646	158063	157873	uncharacterized protein LOC111059587, partial [*Nilaparvata lugens*]
IIV6-WBPH-87	TCONS_00019937	XP_022207646	-	74	2.00E-24	1	74	157963	157891	28S ribosomal protein S7, mitochondrial [*Nilaparvata lugens*]
IIV6-WBPH-88	TCONS_00020862				3.00E-45					
IIV6-WBPH-89	TCONS_00020864	XP_022193248	+	104	4.00E-45	161	264	157984	158087	protein spire-like [*Nilaparvata lugens*]
IIV6-WBPH-90	TCONS_00020863				4.00E-45					
IIV6-WBPH-91	TCONS_00021045	XP_022191335	-	67	2.00E-28	1	67	157963	157897	guanine nucleotide-binding protein subunit beta-like protein [*Nilaparvata lugens*]
IIV6-WBPH-92	TCONS_00021158	No blast hits	-	67	7.00E-27	1	67	157963	157897	No blast hits
IIV6-WBPH-93	TCONS_00022159	No blast hits	+	71	2.00E-25	486	556	158009	158079	No blast hits
IIV6-WBPH-94	TCONS_00023296	No blast hits	-	75	5.00E-30	88	162	157945	157871	No blast hits

IIV6-WBPH-95	TCONS_00023352	XP_022191970	-	93	4.00E-39	528	620	157965	157873	glutamate receptor ionotropic, NMDA 2B [*Nilaparvata lugens*]
IIV6-WBPH-96	TCONS_00023354					518	610			
IIV6-WBPH-97	TCONS_00023659	XP_022189945	+	104	1.00E-42	1	104	157984	158087	aurora kinase C-like [*Nilaparvata lugens*]
IIV6-WBPH-98	TCONS_00024530	XP_022199494	-	70	8.00E-28	473	542	157966	157897	uncharacterized protein LOC111056438 [*Nilaparvata lugens*]
IIV6-WBPH-99	TCONS_00024840	XP_018917922	-	227	7.00E-93	5489	5715	158119	157893	uncharacterized protein LOC109044571 isoform X1 [*Bemisia tabaci*]
IIV6-WBPH-100	TCONS_00025084	No blast hits	-	67	7.00E-29	1	67	157963	157897	No blast hits
IIV6-WBPH-101	TCONS_00025210	XP_022194380	-	69	3.00E-28	1	69	157963	157895	dolichyl-diphosphooligosaccharide--protein glycosyltransferase 48 kDa subunit [*Nilaparvata lugens*]
IIV6-WBPH-102	TCONS_00025415	XP_022195374	-	185	4.00E-69	54	236	158080	157897	LOW QUALITY PROTEIN: polyubiquitin-C-like [*Nilaparvata lugens*]
IIV6-WBPH-103	TCONS_00026241	No blast hits	-	182	5.00E-77	1091	1272	158058	157877	No blast hits
IIV6-WBPH-104	TCONS_00026127	XP_022196604	-	104	7.00E-39	590	693	157979	157876	protein enabled [*Nilaparvata lugens*]
IIV6-WBPH-105	TCONS_00026129				8.00E-39	780	883			
IIV6-WBPH-106	TCONS_00026393	XP_022194702	-	105	2.00E-30	639	735	158000	157896	ribonuclease P protein subunit p29 [*Nilaparvata lugens*]
IIV6-WBPH-107	TCONS_00026444	XP_022199854	+	99	1.00E-14	2379	2474	157985	158082	uncharacterized protein LOC111056767 isoform X2 [*Nilaparvata lugens*]
IIV6-WBPH-108	TCONS_00026590	XP_014602450	+	117	3.00E-44	980	1096	157878	157994	PREDICTED: AN1-type zinc finger protein 2A-like [*Polistes canadensis*]
IIV6-WBPH-109	TCONS_00026483	XP_022207828	-	203	2.00E-86	4797	4999	158124	157922	ral GTPase-activating protein subunit beta [*Nilaparvata lugens*]

IIV6-WBPH-110	TCONS_00026482			257	8.00E-100	4818	5072	158124	157871	
IIV6-WBPH-111	TCONS_00026484		-	257	7.00E-100	4330	4584	158124	157871	
IIV6-WBPH-112	TCONS_00026727	XP_022194578	+	100	3.00E-38	695	794	157984	158083	mitochondrial import inner membrane translocase subunit Tim8 A-like [*Nilaparvata lugens*]
IIV6-WBPH-113	TCONS_00027035	XP_024081474	+	114	4.00E-45	8783	8896	157871	157984	probable chitinase 10 [*Cimex lectularius*]
IIV6-WBPH-114	TCONS_00027690	XP_018497093	-	100	7.00E-43	1	100	157963	157864	PREDICTED: uncharacterized protein LOC108864993 [*Galendromus occidentalis*]
IIV6-WBPH-115	TCONS_00028116	No blast hits	-	67	6.00E-27	1	67	157963	157897	No blast hits
IIV6-WBPH-116	TCONS_00029114	No blast hits	+	100	8.00E-34	178	277	157875	157974	No blast hits
IIV6-WBPH-117	TCONS_00029149	XP_022200041	-	67	2.00E-23	359	425	157941	157875	synaptotagmin-9-like [*Nilaparvata lugens*]
IIV6-WBPH-118	TCONS_00029541	XP_022203432	-	207	1.00E-82	3	209	158103	157897	alpha-tocopherol transfer protein-like [*Nilaparvata lugens*]
IIV6-WBPH-119	TCONS_00029589	XP_022194505	+	118	2.00E-49	1148	1264	157871	157988	uncharacterized protein LOC111052179 [*Nilaparvata lugens*]
IIV6-WBPH-120	TCONS_00029723					1942	2083			
IIV6-WBPH-121	TCONS_00029724					2002	2143			
IIV6-WBPH-122	TCONS_00029722	AQS60680	-	142	4.00E-61	1813	1954	158015	157874	cytochrome P450 SF [*Sogatella furcifera*]
IIV6-WBPH-123	TCONS_00029726					3302	3443			
IIV6-WBPH-124	TCONS_00029725					1809	1950			
IIV6-WBPH-125	TCONS_00029721					2495	2636			

IIV6-WBPH-126	TCONS_00029740	No blast hits	+	76	2.00E-31	1021	1096	157983	158058	No blast hits
IIV6-WBPH-127	TCONS_00030291	XP_022192857	-	80	4.00E-33	231	310	157956	157877	cadherin-86C-like [*Nilaparvata lugens*]
IIV6-WBPH-128	TCONS_00030292				5.00E-33					
IIV6-WBPH-129	TCONS_00030833	XP_022193873	-	118	7.00E-34	1	110	158014	157897	intraflagellar transport protein 140 homolog [*Nilaparvata lugens*]
IIV6-WBPH-130	TCONS_00031118	XP_022193154	+	236	9.00E-18	1731	1941	157874	158108	growth/differentiation factor 8-like [*Nilaparvata lugens*]
IIV6-WBPH-131	TCONS_00031119				1.00E-17	1956	2166			
IIV6-WBPH-132	TCONS_00031041	XP_022195498	-	67	1.00E-24	1	67	157963	157897	uncharacterized protein LOC111052977 [*Nilaparvata lugens*]
IIV6-WBPH-133	TCONS_00031428	XP_022192570	-	148	9.00E-44	3257	3403	158020	157874	tyrosine-protein kinase CSK-like [*Nilaparvata lugens*]
IIV6-WBPH-134	TCONS_00032476	No blast hits	-	247	2.00E-88	24	269	158122	157876	No blast hits
IIV6-WBPH-135	TCONS_00032646	XP_022192184	-	91	2.00E-34	501	591	158125	158035	lysophospholipid acyltransferase 5-like isoform X1 [*Nilaparvata lugens*]
IIV6-WBPH-136	TCONS_00032645					324	414			
IIV6-WBPH-137	TCONS_00033168	XP_022190332	-	138	3.00E-53	1027	1163	158034	157897	uncharacterized protein LOC111048704 isoform X3 [*Nilaparvata lugens*]
IIV6-WBPH-138	TCONS_00033485	XP_022189658	+	71	4.00E-30	2111	2181	157984	158054	uncharacterized protein LOC111048112 isoform X2 [*Nilaparvata lugens*]
IIV6-WBPH-139	TCONS_00033486				7.00E-30	3947	4017			
IIV6-WBPH-140	TCONS_00033487	XP_022189659	+	71	6.00E-30	3907	3977	157984	158054	interaptin-like isoform X3 [*Nilaparvata lugens*]
IIV6-WBPH-141	TCONS_00033557	AIN44119	-	98	6.00E-40	3019	3116	157975	157878	ATP-binding cassette sub-family G member 4-like protein [*Laodelphax striatella*]

IIV6-WBPH-142	TCONS_00034030	XP_022160478	-	67	3.00E-28	1	67	157963	157897	piggyBac transposable element-derived protein 4-like [*Myzus persicae*]
IIV6-WBPH-143	TCONS_00034250	XP_022194702	-	105	3.00E-32	648	744	158000	157896	ribonuclease P protein subunit p29 [*Nilaparvata lugens*]
IIV6-WBPH-144	TCONS_00034251					693	789			
IIV6-WBPH-145	TCONS_00034449	XP_022184245	-	93	7.00E-40	1	93	157963	157871	protein yellow-like [*Nilaparvata lugens*]
IIV6-WBPH-146	TCONS_00034933	XP_022905894	+	120	2.00E-42	1855	1974	157983	158102	uncharacterized protein LOC111417759 [*Onthophagus taurus*]
IIV6-WBPH-147	TCONS_00035379	No blast hits	+	70	2.00E-28	66	135	157982	158051	No blast hits
IIV6-WBPH-148	TCONS_00035852	No blast hits	-	118	5.00E-41	1	110	158014	157897	No blast hits
IIV6-WBPH-149	TCONS_00035564	XP_022201544	+	69	5.00E-28	511	579	157892	157960	uncharacterized protein LOC111058333 [*Nilaparvata lugens*]
IIV6-WBPH-150	TCONS_00035818	XP_022197831	+	41	1.00E-11	698	737	157908	157948	DCN1-like protein 1 [*Nilaparvata lugens*]
IIV6-WBPH-151	TCONS_00035815	XP_022207811	-	122	2.00E-50	1006	1127	158018	157897	osteopetrosis-associated transmembrane protein 1 [*Nilaparvata lugens*]
IIV6-WBPH-152	TCONS_00036302	XP_022199812	+	147	3.00E-57	696	842	157982	158128	uncharacterized protein LOC111056725 isoform X2 [*Nilaparvata lugens*]
IIV6-WBPH-153	TCONS_00036274	XP_022184886	+	249	1.00E-86	2907	3155	157875	158121	uncharacterized protein LOC111044125 [*Nilaparvata lugens*]
IIV6-WBPH-154	TCONS_00036780	No blast hits	-	118	1.00E-51	1	118	158014	157897	No blast hits
IIV6-WBPH-155	TCONS_00037369	XP_022196211	+	92	4.00E-35	1505	1596	157875	157966	lipid droplet-associated hydrolase [*Nilaparvata lugens*]
IIV6-WBPH-156	TCONS_00037368				3.00E-35	1381	1472			

IIV6-WBPH-157	TCONS_00037391					905	1020			
IIV6-WBPH-158	TCONS_00037387					1058	1173			
IIV6-WBPH-159	TCONS_00037390	XP_022187438	+	116	2.00E-48	1544	1659	157984	158099	ATP-dependent DNA helicase Q1-like [*Nilaparvata lugens*]
IIV6-WBPH-160	TCONS_00037388					1055	1170			
IIV6-WBPH-161	TCONS_00037386					1453	1568			
IIV6-WBPH-162	TCONS_00037389					1138	1253			
IIV6-WBPH-163	TCONS_00037628					5857	5936			
IIV6-WBPH-164	TCONS_00037629	XP_022187492	+	80	1.00E-24	5857	5936	157984	158063	transmembrane protein 131 [*Nilaparvata lugens*]
IIV6-WBPH-165	TCONS_00037631					5586	5665			
IIV6-WBPH-166	TCONS_00037630					5798	5877			
IIV6-WBPH-167	TCONS_00037813	XP_022184372	-	114	3.00E-44	1261	1374	158115	158002	maspardin-like [*Nilaparvata lugens*]
IIV6-WBPH-168	TCONS_00037814	XP_022184398	-	114	5.00E-44	2497	2610	158115	158002	2-Cys peroxiredoxin BAS1, chloroplastic-like isoform X3 [*Nilaparvata lugens*]
IIV6-WBPH-169	TCONS_00037835	No blast hits	-	119	8.00E-49	1	119	158014	157897	No blast hits
IIV6-WBPH-170	TCONS_00038572	XP_022200425	-	68	2.00E-28	1	68	157963	157896	neuropeptides capa receptor-like [*Nilaparvata lugens*]
IIV6-WBPH-171	TCONS_00039004	XP_022190772	-	162	3.00E-74	199	360	158057	157896	myelin transcription factor 1-like protein, partial [*Nilaparvata lugens*]
IIV6-WBPH-172	TCONS_00039005					145	306			
IIV6-WBPH-173	TCONS_00039227	XP_022185437	-	94	6.00E-33	1184	1277	158066	157973	uncharacterized protein LOC111044562 [*Nilaparvata lugens*]

IIV6-WBPH-174	TCONS_00040526	XP_022188116	+	142	4.00E-49	277	418	157984	158124	replication factor C subunit 3 [*Nilaparvata lugens*]
IIV6-WBPH-175	TCONS_00040527									
IIV6-WBPH-176	TCONS_00040944	BAQ02368	-	161	2.00E-66	1890	2050	158034	157874	sugar transporter [*Nilaparvata lugens*]
IIV6-WBPH-177	TCONS_00040946					2034	2194			
IIV6-WBPH-178	TCONS_00040990	XP_022187745	-	138	2.00E-60	2260	2397	158034	157897	LOW QUALITY PROTEIN: choline transporter-like protein 1 [*Nilaparvata lugens*]

[1] List of WBPH transcript that mapped to IIV-6 genome; [2] ID of assembled WBPH transcript; [3] GenBank accession number for annotated WBPH transcript; [4] Annotations of assembled WBPH transcript;
